# Unlocking the benefits of transparent and reusable science for climate risk management

**DOI:** 10.1073/pnas.2422157123

**Published:** 2026-01-14

**Authors:** Adam B. Pollack, Lisa Auermuller, Casey D. Burleyson, Jentry Campbell, Madison Condon, Courtney Cooper, Matteo Coronese, Sönke Dangendorf, James Doss-Gollin, Prabhat Hegde, Casey Helgeson, Robert E. Kopp, Jan Kwakkel, Corey Lesk, Justin Mankin, Robert E. Nicholas, Jennie Rice, Samantha Roth, Vivek Srikrishnan, Moira Scheeler, Nancy Tuana, Chris Vernon, Mengqi Zhao, Klaus Keller

**Affiliations:** ^a^Thayer School of Engineering, Dartmouth College, Hanover, NH 03755; ^b^School of Earth, Environment, and Sustainability, University of Iowa, Iowa City, IA 52242; ^c^School of Environmental and Biological Sciences, Rutgers University, New Brunswick, NJ 08901; ^d^Pacific Northwest National Laboratory, Richland, WA 99354; ^e^Dartmouth Libraries, Dartmouth College, Hanover, NH 03755; ^f^Boston University–School of Law, Boston, MA 02215; ^g^Department of Environmental Science and Studies, Berry College, Mt. Berry, GA 30149; ^h^Institute of Economics and L’EmbeDS, Scuola Superiore Sant’Anna, Pisa 56127, Italy; ^i^Department of River-Coastal Science and Engineering, Tulane, New Orleans, LA 70118; ^j^Department of Civil and Environmental Engineering, Rice University, Houston, TX 77005; ^k^Ken Kennedy Institute, Rice University, Houston, TX 77005; ^l^Earth and Environmental Systems Institute, Penn State University, State College, PA 16802; ^m^Department of Philosophy, Penn State University, State College, PA 16802; ^n^Department of Earth and Planetary Sciences and Rutgers Climate & Energy Institute, Rutgers, New Brunswick, NJ 08854; ^o^Faculty of Technology, Policy and Management, Delft University of Technology, Delft, NL 2628 BX; ^p^Department of Geography, Dartmouth College, Hanover, NH 03755; ^q^Neukom Institute for Computational Science, Dartmouth College, Hanover, NH 03755; ^r^Lamont-Doherty Earth Observatory, Columbia University, New York, NY 10964; ^s^Department of Meteorology and Atmospheric Science, Penn State University, State College, PA 16802; ^t^Department of Biological and Environmental Engineering, Cornell University, Ithaca, NY 14853

**Keywords:** climate risk management, climate change, climate impacts, open science

## Abstract

People around the world seek climate risk information to guide their decisions. For instance, projections about future flood risk inform where households choose to live, how lenders manage credit risks, and which communities receive federal funding. Yet data limitations and fundamental validation challenges raise important concerns about the reliability of such projections. The principles of transparency and reusability help address these concerns by enabling scrutiny of assumptions and methods, development of foundational data and tools, and consistent application of evaluation standards. While there is ongoing debate about how much transparency commercial climate risk services should provide, many expect noncommercial actors to lead the way on operationalizing transparency and reusability to fulfill their knowledge-building role in the climate risk ecosystem. However, despite prominent success stories, we find a substantial gap between principles and practice: Only four percent of the most-cited peer-reviewed climate risk studies in recent years fully share their data and code although this is a widely accepted minimum standard for transparency. We highlight low-cost measures that noncommercial researchers can take now to improve transparency and reusability. We also emphasize that transformative progress requires substantial investment, cross-sector collaboration, and careful consideration of tradeoffs, data rights, and multiple perspectives on equity. We hope this perspective accelerates both immediate actions and longer-term conversations to improve the ability of science to effectively support timely, evidence-based, and sound climate risk management.

Climate risk projections increasingly shape high-stakes decisions. For example, millions of prospective US homebuyers used property-level flood risk estimates to guide their search and offers (1). The US government used the same projections in its Climate and Economic Justice Screening Tool to direct billions of dollars to “disadvantaged” communities (2). More broadly, projections about a wide range of physical climate hazards inform decision-making and regulation in many sectors (3).

As demand for these projections grows, so do concerns about their reliability (3–6). A chief concern is that many of these projections claim to have answers to questions beyond what current scientific knowledge can confidently support (7, 8). These projections typically involve three analytical steps: estimating weather probabilities, translating weather to hazards, and estimating damages from hazards. Estimating weather probabilities is the step raising most reliability concerns because of fundamental limitations in verifying models of open, nonstationary systems (9–18). While the openness and nonstationarity of Earth systems are less problematic for the other analytical steps, in practice data limitations constrain our ability to evaluate whether hazard and impact models replicate historical dynamics and observations (19–23).

Consider the property-level flood risk projections that inform millions of homebuyers’ decisions. A recent study comparing two flood hazard models in Los Angeles found only 24% agreement on which properties fall within the present-climate 100-y floodplain (24). Worse, scant historical data on flooding and property damage meant that researchers could not objectively determine which model performed better (24). Even if the authors had adequate observations, substantial challenges remain. For instance, large sampling uncertainties, such as natural climate variability, limit our ability to confirm whether an observed event corresponds to the 100-y floodplain (25–27). Further, hazard is just one driver of risk, and even a hypothetical perfect hazard model does not guarantee precise or accurate risk estimates (28, 29). Of many challenges, even damage models calibrated to observations (which is exceedingly rare) are highly uncertain and generally perform poorly on new events (28–30). These issues, just a subset of considerations in assessing the reliability of property-level flood risk, are further amplified by compound hazards and nonstationarity in risk drivers that produce events with no historical analog (31–33).

A diverse ecosystem of actors works to address these challenges, including government agencies, academic institutions, nonprofits, and commercial entities. Academic and public sector actors play a crucial role in developing foundational methods and knowledge, while commercial and some noncommercial climate services build upon this foundation to provide tailored risk assessments. While there are ongoing debates about who should provide services in different contexts, the foundational knowledge-building role of noncommercial research and data affects the entire ecosystem (3, 34, 35).

Adhering to the principles of transparency and reusability helps noncommercial research fulfill its foundational role. This is critical for confronting verification challenges in climate modeling. Transparency lets others assess assumptions, datasets, and methods. Reusability makes it easier to scrutinize methods, combine models, build on existing work, and run intercomparison projects—all of which are cornerstones of reliable climate knowledge (36–41). The Coupled Model Intercomparison Project (CMIP) exemplifies this approach, coordinating international efforts to answer shared questions while producing data accessible to all to scrutinize, reuse, and build on (40). The benefits of adhering to these principles extend beyond climate projections to hazard and impact estimation as well, with notable examples in ice sheet, sea-level rise, and integrated assessment modeling (36–39, 41).

In fact, transparency and reusability in assumptions, data, and code create benefits that reach far beyond single projects and strengthen the entire climate-risk information ecosystem. When foundational data and models are openly available and reusable, researchers, government agencies, and service providers can make faster, more reliable progress in their respective roles. For example, Nobel laureate William Nordhaus’s Dynamic Integrated Climate-Economy (DICE) model, made openly available with transparent assumptions and reusable code, catalyzed a rich research tradition that continually refines social cost of greenhouse gas estimates and directly informed US EPA regulation (42–49). Decision-makers value this approach for several reasons. First, some climate risk assessments hinge on deeply uncertain elements—climate model choice, damage specifications, discounting, tipping points, and feedbacks—so stakeholders demand transparency to judge whether conclusions are legitimate (8, 35, 43, 50, 51). Second, because regulations and policies can change as knowledge improves and new data becomes available, decision-makers value reusable frameworks that can be updated quickly and reliably (46, 52–54).

Given the high stakes and prominent success stories, one might expect that transparency and reusability are standard in foundational climate risk research. Indeed, many advocates have promoted or standardized these practices (19, 34, 55, 56). For instance, the President’s Council of Advisors on Science and Technology report on extreme weather in a changing climate in 2023 called for open data ecosystems and open-source risk assessments to advance science and support private sector services (19). In terms of standards, funding agencies and high-impact peer-reviewed journals typically require authors to make all data and code available in persistent repositories with unique identifiers (57–60).

Yet as climate risk researchers ourselves, we have observed a disconnect between these stated principles and actual practice. Many of us have produced work falling short of the transparency and reusability principles we recognize as essential. We have also noticed influential climate risk studies, including those widely discussed in popular media, that provide no access to their underlying data or code. This gap between principles and practice led us to assess the state of transparency and reusability in our field.

Our analysis of the most-cited studies in leading climate risk journals from 2021 to 2022 reveals a troubling reality: Only four percent fully share their data and code despite journal transparency requirements (Fig. 1; See *SI Appendix* for details). This lack of openness does not indicate flawed research or in any way undermine the big-picture findings of the climate research community. Still, it shows that our field’s most impactful climate risk research often lacks the transparency and reusability necessary for cumulative building of knowledge through community scrutiny, comparison, and iteration—the very practices that enabled the success stories described above.

**Fig. 1.**
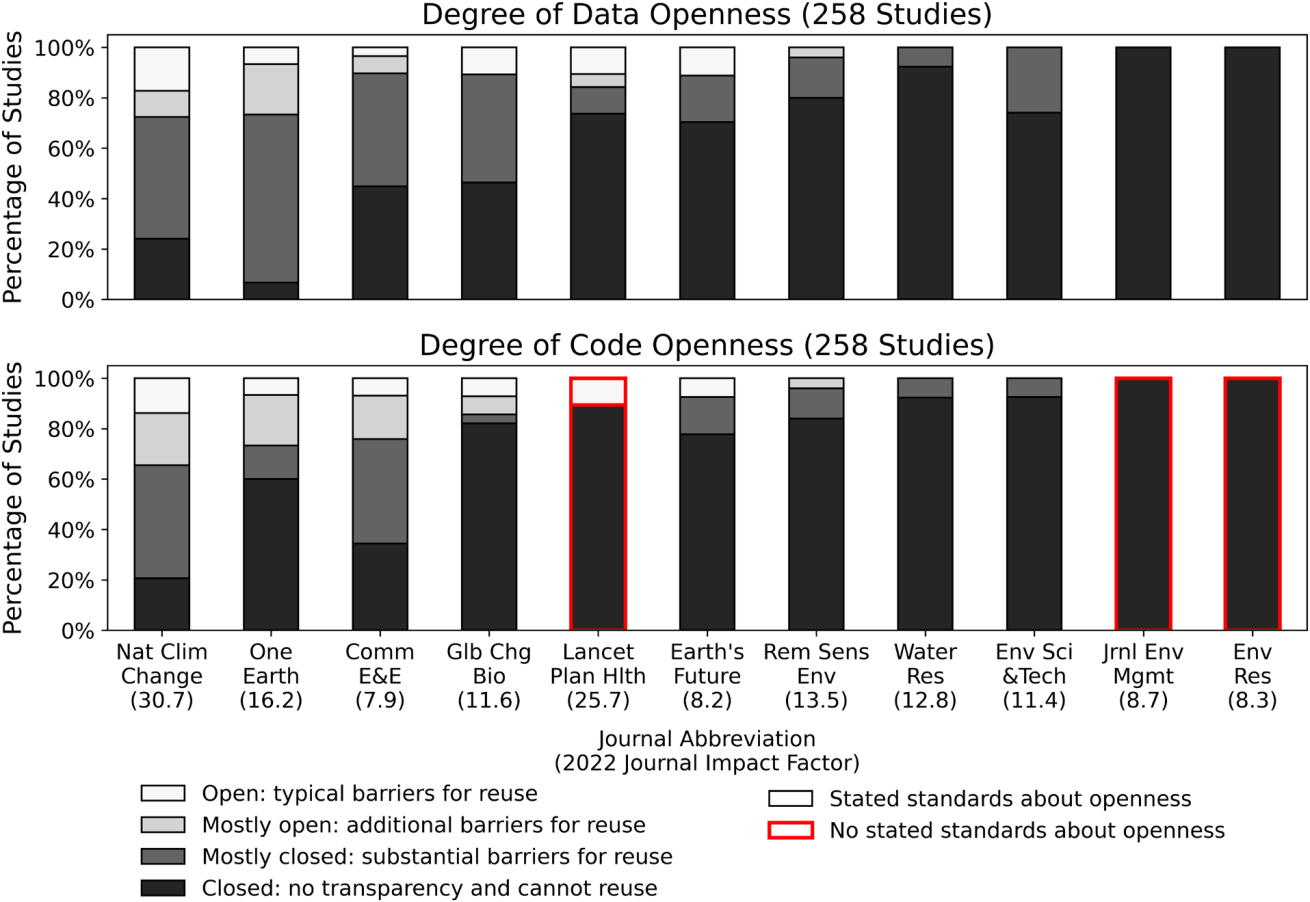
Percentage of the reviewed studies from each journal in our sample that meet different openness standards for data (*Top*) and code (*Bottom*). Journals are ordered along the *x*-axis in terms of the overall proportion of studies published within a journal that make all or most data and code available. Ties in the ranking are broken by journal impact factor. A journal’s 2022 journal impact factor, according to Journal Citation Reports, is represented in parentheses below the journal abbreviation on the *x*-axis. A red border surrounds bars for journals that do not have any stated standards about openness requirements for code (all journals have stated standards about data).

To help address this high-stakes gap, we present an actionable call for change in the academic climate risk research community, moving beyond previous idealistic calls for transparency to address broader systemic challenges in incentives and infrastructure. Our recommendations range from immediate, low-cost actions that individual researchers and journals can implement today to longer-term institutional investments that require careful consideration of tradeoffs and equity. This dual approach acknowledges both the urgency of improving reusability in noncommercial research and the complexity of systemic change.

While we focus on one part of the diverse climate risk information ecosystem, we see this as an urgent and meaningful opportunity that complements ongoing efforts to address broader challenges related to climate risk science, climate services, and their intersection (3, 34, 35). Where relevant, we engage with this broader context to inform future research and discussions. For example, we highlight the need for interpretable AI-based models as well as the foundational role of improved data access in enabling transparency and reusability to deliver on their promised benefits. We hope to inspire our colleagues to improve transparency and reusability in their research, empower a broad audience to hold academic actors accountable for stated standards, and facilitate dialog that can help noncommercial actors better fill their role in the climate risk ecosystem.

## Near-Term Cost-Effective Investments

Strikingly, about 90% of reviewed studies with closed data or code offer no explanation for falling short of journal standards, while only a handful cite challenges to sharing materials such as privacy. Taken together with previously cited success stories and evidence from other disciplines, this pattern points to weak community norms as an important driver of the gap between stated standards and practice (61–65). Strengthening norms entails tradeoffs. In some fields, realigning incentives may yield limited public benefit because their modes of scientific progress and upstream benefits do not clearly hinge on materials sharing and reusability (66–68). In contrast, climate risk research is transdisciplinary, computational-model-centric, and decision-focused. In this setting, transparency and reusability directly address core concerns for information reliability, such as nonstationary Earth systems, incomplete representation of complex processes in models, and the often-limited historical data that constrains model calibration. Thus, as discussed above, adhering to these principles in foundational climate risk research delivers substantial scientific and social value. For these reasons, the large systemic gap we document demands action. Four measures stand out as both necessary and low-cost.

First, journals should enforce their stated standards or update them to ones they will enforce. Journals that fail to do so undermine their credibility and misalign researcher incentives (69). Evaluating material availability, the strictest standards for journals in our sample (*SI Appendix*, Table S1), typically takes reviewers just minutes and should not raise publication fees. Second, regardless of journals’ requirements, researchers should adhere to widely accepted community standards for material availability, such as those from *AGU*’s guidance on data and software availability for authors, which include thoughtful exceptions and require minimal effort (57, 58). The monetary costs of this basic transparency are negligible due to free repositories like Zenodo and Pangeo, and widely popular free technologies such as open-source programming languages, version control systems, and software licensing options (70, 71). There are several cases where full data sharing may be problematic, and we provide guidance on data subset sharing, alternative approaches to facilitating data reusability, and cases where peer review is not appropriate (*SI Appendix*, Boxes S1 and S2).

Third, researchers should cite any used open-source dataset and codebases with digital object identifiers. Citing these resources strengthens incentives and promotes a culture of openness (*SI Appendix*, Box S3). Finally, researchers should write transparent data and code availability statements, especially when some materials cannot be shared (*SI Appendix*, Box S4). Clear statements document valid reasons for limited availability, legitimizing constraints instead of ignoring them, helping other researchers plan related work, and supporting informed editorial decisions. In addition, patterns in availability statements can reveal systemic barriers to transparency and reusability that the scientific community might collectively address.

Going beyond these low-cost transparency measures, low-cost reusability investments can produce net benefits throughout science and society. We illustrate this with the instructive case of “Python for Power System Analysis” (PyPSA). The developers created PyPSA to fill a crucial gap in energy system modeling and invested in transparency and reusability due to the consequential decisions such models inform (72). Less than a decade after its release, the documentation cites 59 diverse universities, research institutes, companies, governments, and nongovernmental organizations that rely on the software for custom applications (73). For example, The Energy and Resources Institute in New Delhi developed “PyPSA-India” for government-supported electricity transition studies, with similar applications across South America, Southeast Asia, and Africa (73–75).

Much of PyPSA’s initial popularity came from its low-cost investments in materials availability, accessible user guides and examples, and clear guidance on how others could contribute (73). These investments enabled the project to evolve into a thriving ecosystem of interconnected open-source initiatives (76). Most notably, PyPSA’s interoperability and reusability initiated the grassroots “PyPSA Meets Earth” project that expanded the ecosystem’s application to global contexts (77). Beyond broadening the spatial scale of impact, this project led to the development of an open-source optimization solver that rivals proprietary alternatives—an advancement with potential applications across multiple disciplines (77, 78).

As PyPSA’s impact hinges on its usability, we suspect that without the developers’ initial investments in reusability community adoption would have occurred more slowly, if at all. For instance, the software’s GitHub repository has over 1,600 stars, 500 forks, and 80 contributors as of August, 2025 (79). The original PyPSA software paper now has over 800 citations according to Google Scholar, and studies with and without the original authors are each cited in the hundreds (72). In contrast, studies show that while research with data and code availability can receive more citations than closed research (61, 80, 81), openness alone is often insufficient for reproduction, let alone for effective reuse (63, 82–86). Thus, the developers’ dedication to materials availability, accessible user guides, and documentation were critical for PyPSA’s research impact. Beyond stalled scientific progress, we worry about the many end-users who use the software and may not be able to afford proprietary alternatives such as research institutes and governments in developing countries.

Importantly, both monetary and time costs for basic reusability are lower than many researchers perceive (65, 69). Even small investments in documentation and testing can dramatically improve reusability (82). Researchers can learn these kinds of “preproducibility” practices (87) in just a few hours [for example, from the Integrated Multisector, Multiscale Modeling (IM3) project’s free “metarepo” template and documentation (88)]. Similarly, free and open-source educational resources, such as MIT’s “CS: Your Missing Semester” and Software Carpentry workshops can also quickly elevate a researcher’s reusability skills (89, 90).

Crucially, in a field where technical validation of projections is severely limited, these reusability investments provide an essential layer of quality assurance. We highlight the critical but arguably overlooked point that these small investments are essential to evaluate most climate risk research for both correctness and understanding. Climate risk research is largely computational, whether in complex modeling or data analysis. These codebases are subject to substantial implementation uncertainties and typically represent only a small subset of plausible ways to perform an inquiry of interest (91–96). Our experience shows that time spent documenting code, writing reproduction instructions, and testing on different machines before sharing with colleagues for reproduction tests more than pays for itself through improved code verification and understanding by colleagues. This sentiment is supported by the praxis and experiences of the global climate modeling community, which invests in reusability practices and experiences lower bug frequency than similarly sized open-source projects despite working with highly complex code bases (97).

## Considering Transformational Investments

We highlight the practices above to emphasize high-return, low-cost investments in transparency and reusability that are achievable and sufficient for many analyses. Individual studies can achieve net benefits from low-cost transparency and reusability investments without investing to the same level of software development and community building as examples like model intercomparison projects, integrated assessment modeling, sea-level rise frameworks, and PyPSA (36, 38–40, 72). However, for the noncommercial climate risk research community to build and sustain more foundational tools that deliver substantial benefits to science and society, we cannot rely solely on grassroots initiatives and low-cost investments. Many prominent success stories in climate-risk research show that influential actors such as funders, research institutions, and professional societies must lead the way (36, 38–40).

Large-scale investments in transparency and reusability can transform the climate risk research community’s ability to advance science and support decision-making. Institutional commitments will involve substantial resource allocation decisions: hiring specialized staff, building and maintaining infrastructure, implementing training programs, and enforcing standards. These would generate many opportunities to enhance research quality, prepare students for diverse career paths and improve the usability of climate risk information. For instance, the NSF-funded INTERSECT program develops research software engineering skills among early-career researchers, creating a pipeline of talent that benefits both academic and commercial sectors (98). However, such actions involve steep opportunity costs and raise important equity concerns about recognition, advantage, and capabilities (68, 99, 100). Rather than offering definitive answers to these complex challenges, we aim to spur broader community dialog by highlighting key considerations that should inform institutional decision-making about transparency and reusability.

An effective public investment strategy must address common data-access barriers that limit even the most transparent and reusable tools. In many climate-risk domains, high-value records, such as address-level flood insurance claims, are inaccessible to researchers due to privacy, administrative, or legal constraints (19, 56). Targeted collaborations between government agencies and academic researchers show that controlled-access arrangements can protect sensitive raw data while producing reusable, decision-relevant outputs (101–104). For example, one recent study used three decades of restricted-access address-level insurance claims to calibrate inundation and damage models, producing both novel insights and a freely available, validated historical flood-extent dataset (102). Such arrangements are uncommon in climate risk research, and the field has not adopted standardized procedures like those used for restricted-access census microdata (105, 106). The result is a shortage of testbeds and benchmarks that would materially improve validation efforts across hazards and models and deliver broad downstream benefits (56).

Institutional investments that expand access to critical datasets can help overcome these constraints and create broader public value. Programs that develop anonymized or synthetic datasets, privacy-preserving data-sharing frameworks, or public benchmarks and testbeds would open the door to faster scientific progress and more innovation and trust in private climate services (19, 56, 107). As emphasized by the President’s Council of Advisors on Science and Technology in its report on extreme weather risk, an open and reusable ecosystem of climate-risk resources provides a foundation upon which skilled service providers can build value-added applications (19). Rather than displacing private innovation, public and reusable data and models enable it—offering benchmarks and core tools that private and nonprofit service providers can customize for specific user needs, as demonstrated by PyPSA, CMIP, and other successful projects (3).

Importantly, institutional investments involve meaningful tradeoffs in resource allocation. For example, when research institutes fund software engineers, legal staff, and other specialized personnel, they forego investment in other areas. Similarly, if funders and journals adopt stricter standards and enforcement beyond simple availability checks and toward deeper reproducibility audits or preregistration-style requirements, research costs will rise. Higher research costs can exacerbate resource allocation tradeoffs and create additional barriers for marginalized groups to participate in science unless paired with targeted support.

We believe that fewer, more transparent and reusable projects ultimately outweigh the benefits of more numerous closed and nonreusable studies. However, institutional actors must account for preexisting resources and incentives when designing new policies. While we are hopeful that automation can make both some reusability practices and stronger enforcement possible without increasing costs, we are wary of stronger standards until low-cost and simple tools exist. Institutions with greater existing resources will navigate new tradeoffs more easily, potentially widening gaps between well-resourced and under-resourced research environments (68, 100). Similarly, early-career researchers face different incentives and constraints than established investigators, and standards that do not account for these differences may disadvantage those most vulnerable in the academic system. For example, early-career researchers trained to use closed-source legacy data or code should not be punished for pursuing career plans that the current academic environment rewards (64).

Institutional actors must also address concerns related to sensitive data and data sovereignty. For example, misused climate-risk projections may lead some actors to make decisions that negatively affect others’ welfare (3). Similarly, respecting Indigenous Peoples’ knowledge about their environments requires specific governance structures and protocols that honor data sovereignty principles (99). These concerns cannot be addressed through one-size-fits-all mandates. The CARE (Collective benefit, Authority to control, Responsibility, and Ethics) and TRUST (Transparency, Responsibility, User focus, Sustainability and Technology) principles complement commonly promoted FAIR (Findable, Accessible, Interoperable, Reusable) principles and offer more nuanced guidance about these situations (99, 108, 109). Approaches such as deidentification, sharing foundational tools without sensitive data, or implementing privacy-preserving computation can balance transparency with protection of individual and community rights (see *SI Appendix*, Box S1 for further discussion) (107, 110, 111).

## Broader Context and Call to Action

While our perspective draws from North American and European contexts, the principles of transparency and reusability resonate globally (112–114). Initiatives like the African Open Science Platform demonstrate how investments in transparency and reusability can serve as an equalizer, enabling underrepresented populations to build capacity, generate credible knowledge, and engage more fully in climate policy discussions (113). This is especially important for developing countries that have contributed least to climate change yet face its most severe impacts and often lack access to the data, models, and technical expertise needed for effective mitigation and adaptation planning (115).

While our perspective focuses on climate risk research, other fields offer valuable models for balancing scientific, public, and commercial considerations when knowledge has practical applications. For instance, the first COVID-19 vaccine was designed within 48 h of the public release of the SARS-CoV-2 genetic code, leveraging decades of publicly funded open research on coronavirus vaccines (116–120). Operation Warp Speed then showed how targeted institutional investment and public–private coordination could complement commercial vaccine development and distribution while respecting proprietary implementations (121, 122). Similarly, the biomedical research community has developed frameworks like the Yale Open Data Access (YODA) project, which enables researchers to access clinical trial data while protecting patient privacy and proprietary interests (123). These cross-sector collaborations generate large social benefits not by abandoning openness, but by pairing a transparent and reusable scientific foundation with targeted public–private roles that effectively balance costs and benefits.

It is important to recognize that transparency, accessibility, and reusability of foundational materials are essential, but they do not fully resolve concerns about producing reliable climate risk information. The publication of commercial AI models in peer-reviewed journals highlights this challenge. For example, Google DeepMind released noncommercial access to AlphaFold3 only after sustained calls from the scientific community, illustrating the synergy between a commercial actor’s pursuit of scientific credibility and the scientific community’s need to scrutinize, extend, test, and interpret the work (124–127). As deep learning models increasingly outperform traditional approaches across a range of applications, including weather and climate prediction, they also raise new interpretability challenges (128–131). Even when models are open-source, their black-box nature can make it difficult for others to understand, extend, and trust (132–134). Still, when these models are open source, they enable others to embark on those efforts (126, 127). As the AlphaFold3 example demonstrates, when foundational materials are made accessible, noncommercial and commercial actors can align: one interpreting, validating, and building trust and the other innovating and disseminating value-added services.

Among many overlapping needs in producing reliable climate risk information, closing current gaps in transparency and reusability within noncommercial climate risk research is both urgent and achievable. We invite others to join us in adopting low-cost practices (see *SI Appendix* for a catalog of our commitments) that improve the transparency, accessibility, and reusability of foundational data, code, models, and knowledge. Doing so strengthens the scientific foundations on which climate risk services depend and ensures that noncommercial actors fulfill their essential role in this rapidly evolving ecosystem.

### Reproducibility Statement.

The computational workflow was tested for reproducibility by Hunter Snyder on November 27, 2023. The reproduction test confirmed that a user who follows the reproducibility instructions posted on the workflow’s repository can successfully reproduce the figures and summary statistics reported in the manuscript.

## Supplementary Material

Appendix 01 (PDF)

## Data Availability

Data and code (abpoll/open: v1.0.1) data have been deposited in Zenodo (https://zenodo.org/records/10291042) (135). Study data are included in the article and/or *SI Appendix*.
