# Supplementary material for: Unlocking the benefits of transparent and reusable science for climate risk management
Source: Proc Natl Acad Sci U S A. 2026 Jan 14;123(3):e2422157123. doi: 10.1073/pnas.2422157123 (PMC12818561; doi:10.1073/pnas.2422157123)
Supplement: Supplementary file 1 — Appendix 01 (PDF) [file pnas.2422157123.sapp.pdf]

The file includes:

Supplementary text

Boxes S1-S7

Table S1

References

Supplementary Text

Low-cost transparency and reusability practices

We - as a group of co-authors focused on climate risk –commit to practices that can have an immediate impact on openness, findability, and accessibility of research materials. While these efforts do not comprehensively address all needed changes, they directly link to important incentive structures such as increased citation and visibility of studies, recognition of open science in grant and tenure review processes, and greater probability that research products will benefit the public (1–4).

As authors, we commit to genuine and traceable efforts for strict data and code openness (Boxes S1, S2). To incentivize these practices across our fields, we commit to citing any open-source dataset or codebase associated with a digital object identifier used in our studies (Box S3). In addition, we commit to sharing transparent data and code availability statements (Box S4).

Box S1: Genuine and traceable efforts for strict data openness.

We commit to making all data associated with peer-reviewed publications open, findable, and accessible to the greatest extent possible. In situations where we depart from full openness, we will justify why these departures are necessary, what steps were taken to overcome data restriction obstacles, and why the level of data openness is justifiable for publication in a peer-reviewed outlet. It is difficult to anticipate every justifiable use case. Instead of proposing rules for every possible use case, we will strive to abide by the principle that with the data and code we provide, future users can reproduce our full computational workflow and check that they get the same (or similar, when processes are stochastic) results as us. Generally, all input data and output data from our analysis will be available, but this is not always possible or ethical. In those cases, demonstrating that users can get the same results on a subset of the data or simulated data is acceptable. We share some *non-exhaustive* use cases below, possibly overlapping, to illustrate which practices we view as acceptable and unacceptable:

1. When input data products are already available in a persistent repository with a unique identifier, it is acceptable and responsible to provide code, or detailed instructions, for downloading this data and structuring it in a project directory (e.g., see Zhao et al. (5) and Ssembatya et al. (6)).
2. When workflows ingest, process, and output large quantities of data (e.g., *AGU* suggests greater than 1 TB (7)), it may be wasteful to post all these resources to a persistent repository with a unique identifier (e.g., sharing all interim data produced while calibrating a large Earth system model). It can be acceptable, practical, and responsible to share a subset of data at each stage of the workflow, and all data at a key stage (e.g., calibrated parameter values or final output data), in a persistent repository with a unique identifier (e.g., see Wong et al. (8)). This

type of use case also addresses cases where input data products are large and not available in a persistent repository with a unique. However, it is not enough to only share the server address or URL to such data since the data may not be persistently stored at this location.

3. When input data products are not public but accessible via license and/or payment, it is acceptable to incorporate this data into a research workflow under some of the following conditions:
    a. The data proprietor uses a license that allows for benchmarking or comparisons (in general or after anonymization or privacy techniques), for example see Nolte et al. (9); and/or
    b. The data proprietor allows for a public release of a subset of this data such that the remaining workflow can be tested for correctness and reuse; and
    c. The data proprietor allows for a license user to provide detailed metadata documentation, a thorough description of quality assurance procedures, and share their input data processing code so that external parties can procure data of the same, or similar, specification for related inquiries (e.g., Nolte et al. (9)).
    d. The data represents a modular part of the system under study (i.e., is not the only input data) and is the only data that is not open, findable, and accessible (e.g., see Deines et al. (10)).
4. When input data products are not public but accessible via license and/or payment, and none of the conditions in point 3 (above) are met, it is worth re-evaluating whether it is appropriate to pursue publication in peer-reviewed journals. Researchers may consider other outlets such as white papers or technical reports. Note that if users of restricted data are not allowed to conduct their own benchmarking or comparisons, the data provider must provide transparent quality assurance themselves. Depending on how transparent and rigorous these assurances are, end-users should communicate that the data have uncertain quality.
5. When raw data products are procured under a confidentiality agreement or pertain to sensitive data with ethical or legal concerns, conduct and document an effort to employ methods with differential privacy or other anonymization techniques with the permission of required parties, such as an institutional review board and survey participants (e.g., see Deines et al. (10)). When this is not possible, provide detailed metadata documentation, a thorough description of quality assurance procedures, and code that processes the raw data such that external parties can procure data of the same specification for related inquiries. It may also be practical and helpful to provide simulated raw data for end-users to conduct reproducibility checks and prepare their workflows for when they obtain appropriate agreements to work with the same or similar data.

Box S2: Genuine and traceable efforts for strict code openness.

We commit to making all code associated with peer-reviewed publications open, findable, and accessible to the greatest extent possible. This includes code for downloading data, processing data, running models, generating results, and producing figures and tables. In situations where we depart from full openness, findability, and accessibility we will justify why these departures are necessary, what steps were taken to overcome code restriction obstacles, and why the level of code openness is justifiable for publication in a peer-reviewed outlet.

When possible, coding in open-source programming languages is preferrable because it allows the broader community to recognize when bugs and biases in the base code may influence research results. However, it is important to acknowledge that researchers are often trained in proprietary programming languages and should not be penalized for these experiences. In this case, findable and accessible code allows others to create open-source counterparts if desired.

Finally, we will avoid code that can only be run by a small group due to model access restrictions. On this point, it is important to acknowledge that some peer-reviewed research on climate impacts employs restricted license proprietary models that are used in current decision-making contexts (e.g., by utility companies). Researching the properties of these models is societally important because of its decision relevance. In many cases, it may be possible to make model code FAIR (and possibly open) while respecting the proprietary nature of data used for decision-making (See Box S1).

Box S3: Explicitly cite all datasets and software that are associated with a digital object identifier such that authors receive credit for reuse.

We commit to citing open-source datasets and tools associated with a digital object identifier such that authors receive credit for our reuse (i.e., citations are reflected on databases such as Web of Science or Google Scholar). This strengthens incentives for open data and code workflows and is a productive way to promote a culture of openness. It is crucial that developers of datasets and computational tools are given appropriate credit for their work. We acknowledge there is some subjectivity in which software use should be cited. Our view is that if a developer requests their software be cited by users, that software should always be cited in the manuscript. For example, any libraries or codebases with associated manuscripts (e.g., models developed for an Environmental Modeling and Software or Journal of Open Source Software publication) should always be explicitly cited in the manuscript.

Box S4: Write transparent data and code availability statements.

We commit to improving the transparency and clarity of data and code availability statements. Data and code availability statements should indicate whether all, some, or none of the data and code is freely open in a persistent repository with a unique identifier. In addition, we will provide extensive details about why missing data or code is not open, findable, and accessible.

Relatedly, we commit to sharing transparent “preproducibility” statements (Box S5), in which preproducibility refers to the principle that a study has been described in adequate detail to allow others to perform the same or a similar study (11). As colleagues, we commit to evaluating the credibility and impact of research articles based on openness and preproducibility (Box S6). In addition, we strive to make ourselves available to perform preproducibility checks for research projects we are not coauthors on (Box S7).

Box S5: Write a transparent preproducibility statement.

We commit to writing transparent preproducibility statements. Preproducibility checks typically consist of (i) evaluating data and code openness, findability, and accessibility; (ii) testing that a code environment configures correctly on a new machine; and (iii) and providing feedback to our colleague about how to clarify project documentation. In our experiences, preproducibility tests are quick to conduct, a strong predictor of reproducibility, improve research documentation, and serve as productive collaboration and training opportunities. Preproducibility statements should define the extent to which an analysis has been tested for preproducibility or even reproducibility (good to strive for, but potentially difficult to secure or unfair to request of colleagues).

Workflow management systems, such as Snakemake, can make these checks a normal part of the research production process (12). Adopting reproducibility frameworks like the meta repository framework from the IM3 project can make these checks a lighter load on colleagues (13, 14).

Box S6: Evaluate credibility and impact conditioned on openness and preproducibility.

We commit to communicating the appropriate caution and uncertainty about the strength of evidence and claims in studies that are not fully open and have not been checked for preproducibility. Going forward, when reviewing articles for peer-reviewed journals that do not meet stated openness standards, we will recommend rejection to the editor. Relatedly, when serving as a reviewer for funding proposals, we will carefully evaluate data management plans with respect to stated agency standards. Finally, we commit to valuing openness and preproducibility when serving on promotion, tenure, or hiring committees.

Box S7: Be available to colleagues for preproducibility checks for research we are not co-authors on.

We strive to foster a culture of reciprocity for openness and preproducibility checks. When it is not a major undertaking to test for reproducibility, we will ask colleagues to perform this more time-consuming and difficult check. For example, we are grateful to a colleague who performed a reproducibility check for the data processing and generation of summary statistics and figures underlying this paper. The colleague also provided notes about how we could improve the clarity of the reproducibility instructions included in our GitHub repository.

Supervisors and mentors can play a substantial role in endorsing and demonstrating the importance of this responsibility. In addition, they can leverage their larger networks to provide these resources for mentees.

More detailed analysis results

We measure degrees of a study's data and code openness using simple metrics. When no data or code is open, findable, or accessible, we use the "closed" category. We classify data and code as "open" when all data and code are open, and findable, and accessible. For studies that share some data or code, we classify studies into two additional categories. "Mostly open" data studies make at least two complete data processes (i.e., input and output data) open, findable, and accessible. "Mostly open" code studies make all analysis code open but not findable or accessible as consistent with FAIR principles (22). A common example of "mostly open" code is when authors provide a code repository link that is not a permanent snapshot of a codebase at the time an analysis was conducted. There are a few cases where the data and code openness, findability, and accessibility are less complete than these examples, but we err on the side of "mostly open" classifications to provide an optimistic estimate.

The final category is “mostly closed.” “Mostly closed” data studies either make only one data process open, findable, and accessible or make some data processes open but not findable or accessible. A common example is when authors make only one input data source (out of many) open, findable, and accessible, provide other input data sources as URLs (often no longer working), and do not provide any interim or output data. “Mostly closed” code studies capture cases in which code is not “closed,” but only one part of code is open, findable, and accessible. A common example is when authors share a link to a GitHub repository for model code, but not code for running the model with their input data or processing and analyzing the model output. Another common example is when authors share only code to generate figures.

Only 10 of the 258 analyzed publications — less than four percent — live up to the widely promoted standard of fully open data and code. This is true even though all journals in our sample state that all data should be shared in a persistent repository with a unique identifier and nearly all journals state similar standards for code (Table S1). Approximately 10 percent of studies in our sample qualify for “mostly open” or “open” code and “mostly closed,” “mostly open,” or “open” data, a charitable estimate of the minimum amount of availability needed to facilitate portability of research methods and results. In stark contrast, 84 percent of our sample is “mostly closed” or “closed” for both data and code.

Of the 228 “closed” or “mostly closed” code studies, 88% provide no explanation for falling short of widely promoted minimal transparency standards. The most common explanation for closed or mostly closed code studies is that code is available upon request (5%) or upon reasonable request (3%). Authors cited proprietary material one time and an inability to share code, with no further explanation, once.

Of the 233 “closed” or “mostly closed” data studies, 89% gave no explanation for why they fall short of minimal standards. The most common explanation is that data is available upon request (6%) or upon reasonable request (4%). Two studies asserted that they did not have permission to share data because of privacy or confidentiality. Two other studies asserted that they did not have permission to share data, but do not state an explanation.

The 10 studies with “mostly open” data, and 17 studies with “mostly open” code, exhibit a variety of departures from widely promoted openness standards. Regarding “mostly open” code, five studies made all their code available in a GitHub repository and three studies provide a subset of code to process input data and generate all model outputs and figures. We emphasize that this level of openness is substantially more open than the “mostly closed” and “closed” code studies and may be sufficiently open and FAIR to promote reuse. However, we also emphasize that these examples represent room for improvement to promote reuse. For example, authors may update GitHub repositories after publication to build on a published study for new projects. In such cases, it may be difficult for other researchers to identify and work from the snapshot of the repository that underlies a published study. This is the reason publishers like *Springer Nature* and

AGU require authors to create a static version of the codebase they used to generate a published study's results (57, 58).

Regarding "mostly open" data, the 10 studies share some combination of their processed data, model output data, and data used in figures and tables, but do not share all their raw data in repositories with unique identifiers. We emphasize that this level of data openness might be sufficient for openness and FAIR-ness – and even desirable. For example, there may be logistical challenges for sharing very large datasets and it may be redundant, wasteful, or not allowed for authors to store data in a persistent repository with a unique identifier that is already available in such a repository or that they did not create. As long as authors provide clear instructions about identifying and downloading data (i.e., not just providing URLs to home pages, which was common in our sample) and structuring a project directory, future users may be able to scrutinize and build on a study if all code is available.

Despite the few "mostly open" edge cases, a key finding is that few studies have even the "mostly open" level of data or code openness. Notably, this is true regardless of peer-reviewed journal standards or journal impact factor (Figure 1). In some journals, not a single highly cited study meets this standard, even though the journal requires it (Table S1). The journal that publishes "open" and "mostly open" research the most often, *Nature Climate Change,* published only 28% and 34% highly cited studies with these categories of data and code, respectively.

Additional information on methods

Our goal was to identify a sample of the "top" climate-risk research publications in the "top" climate change research peer-reviewed scientific journals from recent years, as determined by Journal Citation Reports (JCR), to evaluate whether these influential studies freely share data and code. We intentionally sample articles in this fashion because it is our view that the impact of climate-risk research should be conditioned on its openness. Our sampling strategy does not allow us to comment on the level of data and code openness across climate-risk research publications more broadly.

We obtain our sample of articles as follows. In our subjective view, Nature Climate Change (NCC) is the top climate-focused peer-reviewed journal in terms of reputation. We searched for Nature Climate Change in Journal Citation Reports and looked at the top impact factor journals that fall in its three Journal Citation Reports categories as of June 15, 2022: ENVIRONMENTAL STUDIES - SSCI; METEOROLOGY & ATMOSPHERIC SCIENCES - SCIE; ENVIRONMENTAL SCIENCES - SCIE. The highest impact factor journals are in the ENVIRONMENTAL SCIENCES - SCIE category, and the top 50 are included in the "data/raw/AdamPollack_JCR_JournalResults_09_2023.csv" file that is made available with all of our research materials. Of these journals, we search for the unique publisher name in JCR and add it as a column to a new dataset "data/interim/JCR_SCIE_Filtered.csv." We sort the journals based on 2022 JIF, according to JCR, and retain the highest JIF journal for each publisher. We also do not include journals that publish exclusively review articles. This leaves us with a list of 18

journals. Of these, we search Web of Science with the following search string: ((IS=journal ISSN or eISSN) AND (TS=clim*) AND ((PY=2021) OR (PY=2022)), adding the appropriate ISSN for each journal, and narrow results using filters to limit item type to article, and years of publication to 2021 and 2022. We use the topic term "clim*" to restrict our search to journal articles with variations on climate in the title, abstract, or keywords, ensuring that climate is a core topical aspect of the article. We sort the results from most to least cited and download the first 30 entries as a tab delimited file. Each article is inspected for being a research article with original materials, having a climate change focus, and receiving at least 20 citations since publication. All journals with at least ten articles that meet these criteria are considered sufficiently impactful for inclusion in our review.

Our sample spans 258 articles from 11 journals. We record the following fields for each article:

1. is_climate: 0/1 for climate change focus. The main idea is to "drop" studies which may be in our sample because they use climatic factors or variables but are not studying physical processes of climatic change and/or do not invoke climate change or climate risk management as a motivation for the study
2. dropped: 0/1
3. dropped_why: not climate focused, no data or code (if perspective, for example)
4. data_statement: copy/paste data statement
5. code_statement: copy/paste code statement
6. data_open: Open, Partially Closed, Closed.
    a. If a study shares data in a pdf, such as in a table, it is generally not machine readable and is considered closed (not partially closed). This is almost certainly not the way the data was stored and extracted for the analysis conducted in the paper.
    b. If a study shares code to download all raw data, this can count as open data.
    c. If a study stores everything in a permanent repository but users need to request access, this is not open. Unless some data is available on the repository without restriction, this is classified as closed.
7. code_open: Open, Partially Closed, Closed.
    a. Open refers to the best practice of all code available in a persistent repository with a unique identifier.
    b. Partially closed captures any instance that is not open or closed.
8. data_repo: Where is data stored? ";" delimited for multiple. Can be URLs, GitHub, Zenodo, etc. Only some of these are permanently and persistently archived & free.
9. code_repo: Where is code stored? ";" delimited for multiple. Can be with the paper, GitHub, Zenodo, etc. Only some of these are permanently and persistently archived & free
10. data_included: ";" delimited for which data is included. Is "none" for "Closed" data, and "raw," "processed," etc. for partially closed (if any of the data is available from some category, add it to this list even if some of the data from that category is missing), and "all" for "Open"

    a. Important to note that just because “Raw” is named doesn’t mean all raw data is included. It is often too hard to tell without the author explicitly stating all data is available. “Raw” means data that partially corresponds to FAIR (accessible, machine readable) is provided. If there is no permanently available data referenced in the data availability statement, it’s closed. So, providing just URLs is closed.
    b. If you share code to download all raw data, this counts as raw data being included. A more careful check would examine whether the download links point to a unique and persistent repository.
11. data_limitation_other: “;” delimited for more information about what is included in a data availability statement. For example, URLs provide more context for issues when it may seem like raw data is provided but it’s actually closed.
12. code_included: analogously defined to data_included.
13. data_reasons: Any explanations for missing data
14. code_reasons: Any explanations for missing code
15. notes: some articles called for some extra detail to justify coding decisions

As discussed in the main text, we classified studies into four categories for degrees of code or data openness. These classifications correspond to combinations of the fields data_open and data_included for degrees of data openness and code_open and code_included for degrees of code_openness.

A. Data categories
    a. Open: data_open is “Open”
    b. Mostly open: data_open is “Partially closed” and data_included is one of “All,” “Raw; Results; Source Data”, or “Raw; Results.” The reason “All” data_included corresponds to “Partially closed” is that not all of the data was stored in a persistent repository with a unique identifier.
    c. Mostly closed: data_open is “Partially closed” and data_included is not one of the categories from b)
    d. Closed: data_closed is “Closed.”
B. Code categories
    a. Open: code_open is “Open”
    b. Mostly open: code_open is “Partially closed” and code_included is one of “All,” “Download; Process; Analysis; Figures,” “Processing; Generate Results,” “Processing; Results,” “Models; Results,” “Models; Analysis,” “Analysis; Figures,” “Model; Figures,” “Results; Figures,” and “Generate Results; Figures.” The reason “All” code_included corresponds to “Partially closed” is that not all of the code was stored in a persistent repository with a unique identifier.

c. Mostly closed: code_open is "Partially closed" and code_included is not one of the categories from b)
d. Closed: code_closed is "Closed."

Supplementary Tables

Table S1. References supporting the data and code stated standards coding. All statements were last accessed from the listed URLs on November 17, 2023.

Journal	Open Data Standards	Open Code Standards	URL
Nature Climate Change	Yes. For example [emphasis theirs], "a condition of publication in a Nature Portfolio journal is that **authors are required to make materials, data, code, and associated protocols promptly available to readers without undue qualifications**."	Yes. See left column.	https://www.nature.com/nature-portfolio/editorial-policies/reporting-standards
Communications Earth & Environment	Yes. See above.	Yes. Same as above.	https://www.nature.com/nature-portfolio/editorial-policies/reporting-standards
Earth's Future	Yes. For example, "Primary and processed data used for your research should be preserved and made available."	Yes. For example, "authors should make available software that has a significant impact on the research."	https://www.agu.org/publish-with-agu/publish/author-resources/data-and-software-for-authors
Lancet Planetary Health	Yes. For example, the "Data sharing" section of the info for authors document explains "from September 21, 2020, all submitted research Articles must contain a data sharing statement, to be included at the end of the manuscript," but there is no indication about what degree of data availability is encouraged or required.	No, to the best of our knowledge.	https://www.thelancet.com/pb-assets/Lancet/authors/tlplanet-info-for-authors.pdf

Global Change Biology	Yes. For example, "*Global Change Biology* requires, as a condition for publication, that the data supporting the results in the paper be archived in an appropriate public repository. Data archiving must be completed before files will be sent to the publisher. Both primary *and* secondary data needs to be publicly available as a condition for publication."	Yes. For example, "*Global Change Biology* mandates the sharing of code, software and documentation supporting the results in the paper following acceptance. It may be archived in an appropriate public repository or made accessible from a long-term server (e.g., GitHub)."	https://onlinelibrary.wiley.com/page/journal/13652486/homepage/forauthors.html
One Earth	Yes. For example, "As a Cell Press author, you must be willing to share all of the data and original code you report in your published paper with the research community unless there is a countervailing legal or ethical prohibition (e.g., the data are confidential medical records). Your paper must include a comprehensive and accurate "data and code availability" statement that describes the steps you have taken or will take to make your data and original code available after publication. We strongly recommend that you archive all of the unprocessed data and original code that your paper reports in an online repository that meets the criteria for digital longevity, implementation of FAIR standards, and community support as articulated by Fairsharing.org and collaborators. Note that should issues arise after publication, failing to produce your data can be grounds for retraction."	Yes. See entry to the left.	https://www.cell.com/one-earth/author

Remote Sensing of Environment	Yes. For example, "This journal encourages and enables you to share data that supports your research publication where appropriate, and enables you to interlink the data with your published articles."	No, to the best of our knowledge.	https://www.sciencedirect.com/journal/remote-sensing-of-environment/publish/guide-for-authors
Water Research	Yes. For example, "This journal requires and enables you to share data that supports your research publication where appropriate, and enables you to interlink the data with your published articles."	Yes. For example, "Where existing software is used to analyze the data, the software version, computing platform, and parameter information used to run the program should be included in the manuscript or supplemental material and the software should be appropriately cited. In cases where custom software is developed to process data, this software should be published in a general data (e.g., Zenodo, Dryad, institutional repository) or code repository under a license approved by the Open Source Initiative (https://opensource.org/licenses). The associated persistent identifier (e.g., a DOI) should be provided in the manuscript. The software should be accompanied by information about requirements (e.g. computing platform, code libraries) and instructions about its installation and usage, including the clear identification of the input data and its format."	https://www.sciencedirect.com/journal/water-research/publish/guide-for-authors
Environmental Science & Technology	Yes. For example, "*ES&T* requires for all published articles that authors make materials, data,	Yes. For example, "When computer codes are developed or used and are	https://publish.acs.org/publish/aut

	and protocols available to readers through deposition in a public database”	an essential part of a manuscript, sufficient detail must be given, either within the paper or in the SI. Types of languages that are used in the computer codes, compiler/interpreter, and operating system with a specific version must be provided or properly cited.”	hor_guidelines?coden=esthag
Journal of Environmental Management	Yes. For example, “This journal requires and enables you to share data that supports your research publication where appropriate, and enables you to interlink the data with your published articles.”	No, to the best of our knowledge.	https://www.sciencedirect.com/journal/journal-of-environmental-management/publish/guide-for-authors

References

1. National Academies of Sciences, Engineering, and Medicine, *et al.*, *Reproducibility and Replicability in Science* (National Academies Press, 2019).

2. M. R. Munafò, *et al.*, A manifesto for reproducible science. *Nat Hum Behav* **1**, 0021 (2017).

3. M. A. Edwards, S. Roy, Academic Research in the 21st Century: Maintaining Scientific Integrity in a Climate of Perverse Incentives and Hypercompetition. *Environ. Eng. Sci.* **34**, 51–61 (2017).

4. J. Flier, Faculty promotion must assess reproducibility. *Nature* **549**, 133 (2017).

5. M. Zhao, *et al.*, Repository for Zhao et al., 2023 - Geoscientific Model Development. [Preprint] (2023). Available at: https://zenodo.org/records/10211057 [Accessed 27 September 2024].

6. H. Ssembatya, *et al.*, *IMMM-SFA/ssembatya-etal_2024_earths_future: v1.0.0 Release* (Zenodo, 2024).

7. P. Fox, *et al.*, *Data and Software Sharing Guidance for Authors Submitting to AGU Journals* (2021).

8. T. E. Wong, *et al.*, MimiBRICK.jl: A Julia package for the BRICK model for sea-level change in the Mimi integrated modeling framework. *J. Open Source Softw.* **7**, 4556 (2022).

9. C. Nolte, *et al.*, Data Practices for Studying the Impacts of Environmental Amenities and Hazards with Nationwide Property Data. *Land Econ.* (2023). https://doi.org/10.3368/le.100.1.102122-0090R.

10. J. M. Deines, R. Patel, S.-Z. Liang, W. Dado, D. B. Lobell, A million kernels of truth: Insights into scalable satellite maize yield mapping and yield gap analysis from an extensive ground dataset in the US Corn Belt. *Remote Sens. Environ.* **253**, 112174 (2021).

11. P. B. Stark, Before reproducibility must come preproducibility. *Nature* **557**, 613 (2018).

12. F. Mölder, *et al.*, Sustainable data analysis with Snakemake. *F1000Res.* **10**, 33 (2021).

13. C. R. Vernon, C. Burleyson, J. Rice, M. Zhao, MSD CoP Webinar: Using Meta-Repositories To Facilitate Open Science in MSD Research. (2024).

14. C. R. Vernon, E. Rexer, metarepo: A single point of access meta-repository that guides others in how to reproduce an experiment. https://doi.org/10.5281/zenodo.12100494. Deposited 2024.
